# Cochlear Homocysteine Metabolism at the Crossroad of Nutrition and Sensorineural Hearing Loss

**DOI:** 10.3389/fnmol.2017.00107

**Published:** 2017-04-25

**Authors:** Teresa Partearroyo, Néstor Vallecillo, María A. Pajares, Gregorio Varela-Moreiras, Isabel Varela-Nieto

**Affiliations:** ^1^Departamento de Ciencias Farmacéuticas y de la Salud, Facultad de Farmacia, Universidad CEU San PabloMadrid, Spain; ^2^Departamento de Fisiopatología y del Sistema Nervios, Instituto de Investigaciones Biomédicas Alberto Sols, Consejo Superior de Investigaciones Científicas (CSIC-UAM)Madrid, Spain; ^3^Centro de Investigación Biomédica en Red de Enfermedades Raras (CIBERER), Instituto de Salud Carlos IIIMadrid, Spain; ^4^Investigación en Otoneurocirugía, Instituto de Investigación Sanitaria La Paz (IdiPAZ)Madrid, Spain

**Keywords:** folic acid, omega-3, nutritional imbalance, one-carbon metabolism, oxidative stress, rare diseases

## Abstract

Hearing loss (HL) is one of the most common causes of disability, affecting 360 million people according to the World Health Organization (WHO). HL is most frequently of sensorineural origin, being caused by the irreversible loss of hair cells and/or spiral ganglion neurons. The etiology of sensorineural HL (SNHL) is multifactorial, with genetic and environmental factors such as noise, ototoxic substances and aging playing a role. The nutritional status is central in aging disability, but the interplay between nutrition and SNHL has only recently gained attention. Dietary supplementation could therefore constitute the first step for the prevention and potential repair of hearing damage before it reaches irreversibility. In this context, different epidemiological studies have shown correlations among the nutritional condition, increased total plasma homocysteine (tHcy) and SNHL. Several human genetic rare diseases are also associated with homocysteine (Hcy) metabolism and SNHL confirming this potential link. Accordingly, rodent experimental models have provided the molecular basis to understand the observed effects. Thus, increased tHcy levels and vitamin deficiencies, such as folic acid (FA), have been linked with SNHL, whereas long-term dietary supplementation with omega-3 fatty acids improved Hcy metabolism, cell survival and hearing acuity. Furthermore, pharmacological supplementations with the anti-oxidant fumaric acid that targets Hcy metabolism also improved SNHL. Overall these results strongly suggest that cochlear Hcy metabolism is a key player in the onset and progression of SNHL, opening the way for the design of prospective nutritional therapies.

## Hearing Loss

According to the World Health Organization (2015), moderate-to-profound Hearing loss (HL) affects 360 million people worldwide. Its incidence varies in each population segment, affecting already ~10% of children and increasing to 30% of the population over 65 years (Roth et al., 2011; Li-Korotky, 2012). In addition, HL has been ranked as the fifth leading cause of years lived with disability in the Global Burden of Disease Study 2013 Collaborators (2015). Thus, this impairment certainly limits the quality of life, and significantly increases the risk of dependance. Therefore, the identification of factors involved in HL is key to understand the physiopathology, to improve diagnosis and to develop appropriate therapies and preventive behaviors.

The ear consists of three parts of which the cochlea, in the inner ear, is responsible for the mechanotransduction of the sound stimulus. At the cochlea, in the *scala media*, more than a dozen interconnected cell types are fundamental for hearing. The auditory sensory epithelium, the organ of Corti, contains two types of hair cells, outer and inner, innervated by the spiral ganglion neurons that connect it with the brain (Magariños et al., 2012, 2014). The irreversible loss of hair cells and/or neurons, or their malfunctions are the typical causes of sensorineural HL (SNHL).

SNHL has a multifactorial origin that combines genetic with environmental factors (Dror and Avraham, 2009; Roth et al., 2011). Genetic factors comprise mutations in genes or regulatory elements involved in the development, structure or function of the cochlea. For example, a fundamental role in hearing is played by proteins involved in cell-cell junctions (tight junctions, adherent junctions and gap junctions), and among them the connexins. Mutations in five of the 21 genes of the human connexin family have been linked to the onset of deafness (*C*x26, *Cx30*, *Cx31, Cx32* and* Cx43*; Dror and Avraham, 2009). Moreover, knockdown of one of these genes, precisely the *Cx30*, correlates with alterations in cochlear homocysteine (Hcy) metabolism (Cohen-Salmon et al., 2007). Conversely, hyperhomocysteinemia (HHcy) increases the levels of Cx43 in a variety of cellular contexts (Li et al., 2002; Boot et al., 2006). Interestingly, Cbs heterozygous mice show HHcy and impaired matrix remodeling in the cochlea (Kundu et al., 2009). Cell-cell contacts are essential for hearing and, therefore, Hcy accumulation could have deleterious effects on the hearing receptor machinery. Further studies are needed to fully understand the role of Hcy and its metabolites in the cochlea. On the other hand, environmental factors include exposure to high levels of noise, ototoxic drugs or nutritional deficiencies (Gok et al., 2004; Tabuchi et al., 2011). Moreover, certain genetic factors predispose to suffer damage due to noise or ototoxic drugs, as well as, to premature aging of the hearing structures (Zhao et al., 2004; Rydzanicz et al., 2010); their identification is one of the current challenges in this field of research. Approximately 50% of the cases of hereditary SNHL are accompanied by other clinical symptoms, and statistically 1 in every 1000 newborns has profound deafness and one more will suffer HL before adulthood. Altogether, these data suggest that manipulation of micronutrients could be a tool to understand the genes and physiopathological mechanisms involved in hearing and SNHL.

## Hyperhomocysteinemia and Neurosensorial Hearing Loss

HHcy is an acquired metabolic problem that was first described by McCully (1969), and whose interest increased greatly due to the detection of high plasmatic levels of Hcy (tHcy) in cardiovascular diseases (Ueland and Refsum, 1989). Similarly, HHcy also shows a strong correlation with the development of neurological disorders, chronic kidney disease, osteoporosis, gastrointestinal disorders, cancer and the presence of certain congenital defects (Givvimani et al., 2012; Schalinske and Smazal, 2012; Iacobazzi et al., 2014; Lehotsky et al., 2015; Perna and Ingrosso, 2016). In fact, high levels of tHcy are quite common, and are detected in 10%–20% of the population as a result of genetic (Brosnan et al., 2004) and other factors (Refsum et al., 1989; Noga et al., 2003), but their incidence depends widely on geography, age, sex and ethnicity (Yang et al., 2014).

**Figure 1:**
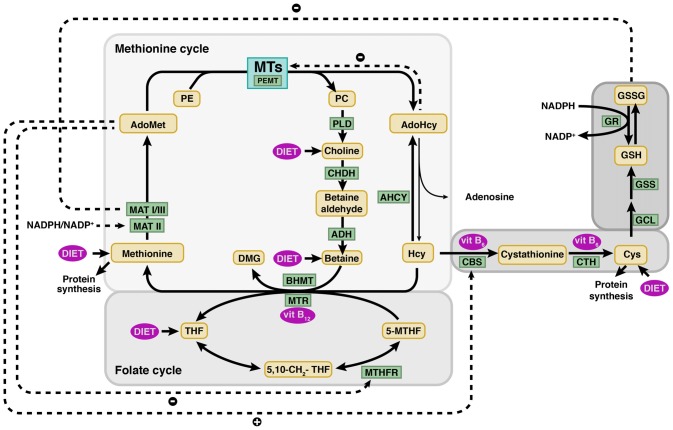
**Methionine and folate metabolism and connecting pathways.** Schematic view of the main metabolic reactions involved in homocysteine (Hcy) metabolism, highlighting the major regulatory mechanisms (dotted lines). Hcy remethylation is part of the methionine cycle and is catalyzed by the vitamin B_12_ dependent methionine synthase (MTR) or betaine homocysteine methyltransferase (BHMT), enzymes that generate methionine using 5′-methyltetrahydrofolate (5-MTHF) and betaine as methyl donors, respectively. Methionine adenosyltransferases (MATs: MAT I, II and III) use methionine to synthesize S-adenosylmethionine (AdoMet). Donation of the AdoMet methyl group renders S-adenosylhomocysteine (AdoHcy) that is hydrolyzed by S-adenosylhomocysteine hydrolase (AHCY) to produce Hcy and adenosine in a reversible reaction. Hcy catabolism takes place initially by serine conjugation, a reaction catalyzed by cystathionine β-synthase (CBS) and that lead to cystathionine synthesis. This metabolite is then utilized by cystathionine γ-lyase (CTH) to produce cysteine (Cys). Both reactions depend on pyridoxal phosphate (vitamin B_6_). The correct function of these pathways depends on a continuous supply of nutrients, methionine, vitamins B_12_ and B_6_ and folate. The latter is used in the folate cycle for the synthesis of 5-MTHF catalyzed by methylenetetrahydrofolate reductase (MTHFR). A reduced ingestion of the aforementioned nutrients leads to a decrease in the flux through these pathways, in which many cellular key compounds are generated (phospholipids, neurotransmitters, etc.). Enzymes and metabolites appear in square and rounded boxes, respectively. ADH, aldehyde dehydrogenase; CHDH, choline oxidase; 5,10-CH_2_-THF, 5,10-methylenetetrahydrofolate; DMG, dimethylglycine; GCL, glutamate-cysteine ligase; GR, glutathione reductase; GSH, reduced glutathione; GSS, glutathione synthase; GSSG, oxidized glutathione; MTs, methyltransferases; NADP^+^, nicotinamide adenine dinucleotide phosphate; NADPH, reduced form of NADP^+^; PC, phosphatidylcholine; PE, phosphatidylethanolamine; PEMT, phosphatidylethanolanime N-methyltransferase; PLD, phospholipase D; THF, tetrahydrofolate; vit B_6_, vitamin B_6_; vit. B_12_, vitamin B_12_.

Regarding hearing impairment and HHcy, several epidemiological studies have shown an association between certain nutritional deficiencies and development of SNHL. Reduced folic acid (FA) concentrations have been found in age-related HL (ARHL) and sudden SNHL, this decrease correlating with either reduced vitamin B_12_ (Houston et al., 1999; Lasisi et al., 2010; Karli et al., 2013) or increased tHcy levels (Cadoni et al., 2004). Although Hcy concentrations reflect alterations in its metabolism (Figure 1), which include the methionine and folate cycles, as well as the transsulfuration pathway, no consistent association between mutations in genes of these pathways and SNHL has been reported (Durga et al., 2006; Uchida et al., 2011). Analysis of the putative relationship between the *methylenetetrahydrofolate reductase (MTHFR)* C677T mutation and ARHL rendered contradictory results (Durga et al., 2006), since this allele has been correlated with hearing impairment and HHcy, and also with a reduced risk of ARHL independent from folate and Hcy levels (Uchida et al., 2011; Fusconi et al., 2012).

Epidemiological research has also provided evidence associating atherosclerosis in the inner ear and poor hearing, and connecting risk factors of vascular disease and ARHL (Rosen and Olin, 1965; Johnsson and Hawkins, 1972; Makishima, 1978). Among them, Hcy is also an agonist of N-methyl-D-aspartate receptors, which are overexcited in SNHL (Puel et al., 1991; Lipton et al., 1997). Thus, the mechanisms linking SNHL and Hcy metabolism seem to include a large variety of pathways, their connections remaining underexplored.

Hcy emerges as a node of key pathways of the intermediary metabolism that has been mainly explored in the liver (Figure 1), whereas knowledge of these routes and their regulation has been scarcely analyzed in peripheral or neurosensory organs (Pajares and Pérez-Sala, 2006; Obeid, 2013). In fact, the cochlea is one of the few sensory organs in which a whole expression and protein profile of the methionine cycle and transsultfuration pathway has been reported (Martínez-Vega et al., 2015a). Based on the hepatic knowledge, attempts to decrease the systemic tHcy levels by the design of several supplementation studies have been carried out. These randomized trials demonstrated the lowering of tHcy levels by dietary folate supplementation (Jacques et al., 1999), and provided the basis for an intervention trial carried out by Durga et al. (2007) to assess effects of folate supplementation on ARHL. The results obtained showed a slower progression of ARHL in individuals receiving the supplement.

SNHL has been associated with rare diseases involving alterations in Hcy levels. This is the case of combined methylmalonic acidemia and homocystinuria cblC type (OMIM 277400), caused by mutations in the *MMACHC* gene. These mutations result in a decreased production of cofactors for methylmalonyl-CoA mutase (adenosylcobalamin) and methionine synthase (MTR; methylcobalamin) and, in turn, in elevated Hcy levels in the cerebrospinal fluid that correlate with unilateral SNHL (Harding et al., 2003; Tsai et al., 2007; Carrillo-Carrasco et al., 2012). Another rare disease reported to result with SNHL and homocystinuria is spastic quadriplegia, retinitis pigmentosa and mental retardation (OMIM 270950; Gordon et al., 1976), but this consanguinity disorder has been described in a very limited number of cases, and hence, the common molecular bases remain unknown. Among patients with osteogenesis imperfecta type I (OMIM 166200), which present with mutations in several genes including *COL1A1* and *COL1A2*, that codify for the α1 and α2 chains of type I collagen, a correlation with SNHL has been also detected from the age of 20-years onward (Hartikka et al., 2004). Hcy is known to decrease the H3K9me2 content on the *COL1A1* gene promoter leading to its upregulation. This effect is dependent on Hcy inhibition of G9a histone methyltransferase expression, which in turn results in lower G9a binding to the neuron-restrictive silencer element (NRSE) of this promoter (Lei et al., 2015). Additionally, Hcy has been also shown to increase oxidative mechanisms and activation of mitochondrial matrix metalloproteinase causing bone matrix degradation and alterations in its biomechanical properties (Behera et al., 2016), facts that could affect mechanotransduction of the sound in the middle ear.

Further studies in animal models confirmed the association between SNHL and HHcy. For example, *Cbs*^+/–^ heterozygous mice show moderate SNHL, high levels of Hcy in the *stria vascularis* and the spiral ligament, oxidative stress and reduction of vessel density, effects that were prevented by the administration of FA in the drinking water (Kundu et al., 2012). In *Cx30*^−/−^ null mice hearing impairment was also associated with alterations in the *stria vascularis*, precisely down- and up-regulation of *Betaine homocysteine methyltransferase (BHMT)* and *Ahcy* expression, respectively, and increased Hcy immunostaining (Cohen-Salmon et al., 2007). All these data, epidemiological and derived from human and mouse genetic studies, reinforced the idea of the importance of Hcy metabolism in deafness.

## Nutritional Interventions and Hearing Loss

To date, supplementation and nutrient deficiency studies have been conducted using laboratory animals, in which auditory thresholds, internal ear microvasculature, or inflammation have been evaluated (Schuknecht et al., 1974; Makishima, 1978); but only a few have analyzed Hcy metabolism following the lead of the epidemiological data (Houston et al., 1999; Cadoni et al., 2004; Lasisi et al., 2010). Two studies in mouse models have shown the relationship between Hcy metabolism and SNHL and the impact of folate deficiency (FD; Martínez-Vega et al., 2015a, 2016). C57BL/6J mice, a mouse strain prone to SNHL, showed increased hearing thresholds after 2 months on a FD diet (Martínez-Vega et al., 2015a). This reduced intake of folate caused decreased serum concentrations of this micronutrient and increased tHcy. These systemic alterations correlated with SNHL, which also coincided with changes in expression and protein levels of cochlear Hcy metabolism aimed at decreasing the production of this amino acid, avoid or moderate its remethylation and catabolism, and induce its elimination into the plasma (Martínez-Vega et al., 2015a).

However, these cellular efforts to balance intracellular levels of Hcy are not sufficient as increased protein N-homocysteinylation was detected in cochlear whole extracts; such post-translational modification may contribute to the inactivation and aggregation of proteins (Sharma et al., 2015). The reduced Hcy utilization by the transsulfuration pathway has an additional negative effect by decreasing H_2_S production via CBS and cystathionine γ-lyase (CTH). The protective role of this gasotransmitter as cochlear vasodilator has been reported in noise-induced HL (Li et al., 2011), and hence, the putative contribution of its reduced levels to the changes described in the microvasculature (Prazma et al., 1990; Gratton and Schulte, 1995). These changes are accompanied by a moderate increase in cochlear oxidative stress, as it is usually the case in the aging process. Altogether, these findings demonstrate that the relationship between HHcy induced by FD and premature SNHL involves cellular degeneration, impairment of cochlear Hcy metabolism and associated oxidative stress (Martínez-Vega et al., 2015a). Moreover, these results were later confirmed in a long-term study on the effects of FD using a mouse strain with delayed SNHL onset, in which, again, the deficiency caused premature SNHL (Martínez-Vega et al., 2016). The latter work also evidenced that the consequences of nutritional imbalances are strongly dependent on the mouse genetic background. Parallel evidences supporting the importance of nutrition on HL come from studies using injection of rodents with a variety of antioxidants, which showed different degrees of otic protection (Fetoni et al., 2010, 2014, 2015). On the other hand, it should be mentioned that an overdose of anti-oxidants may have undesired secondary consequences (Mancuso, 2015).

**Figure 2:**
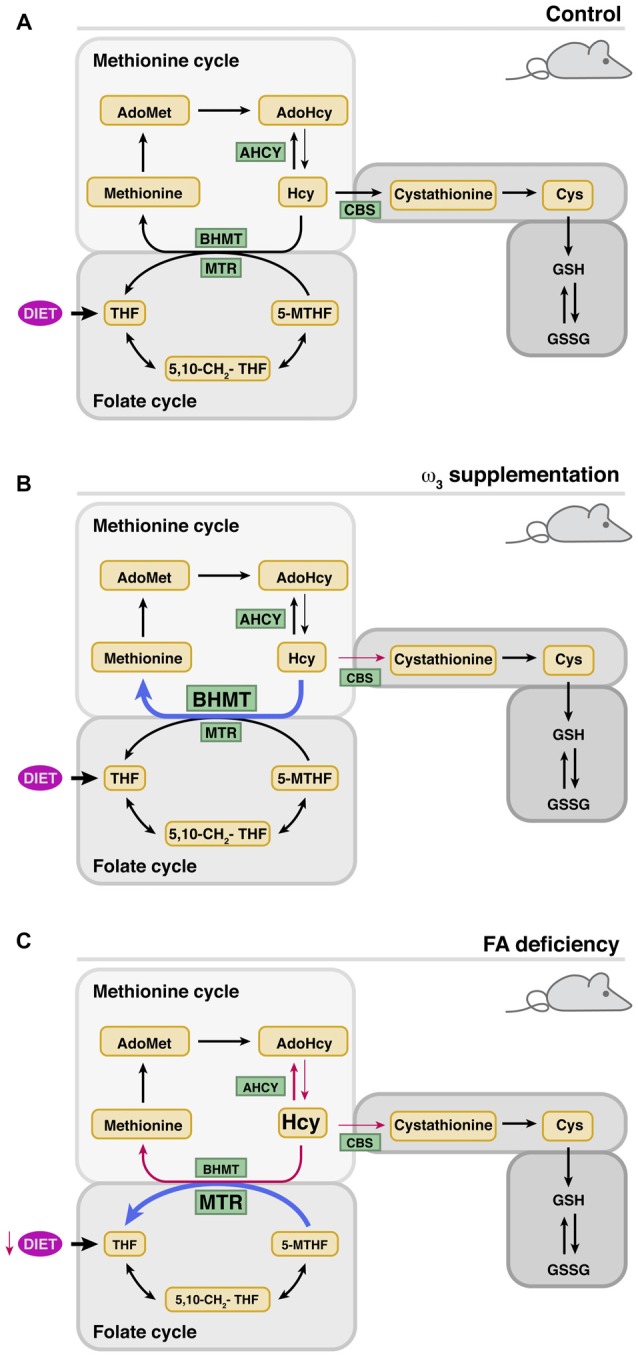
**Modulation of cochlear metabolism of Hcy by the diet.** The thickness of the arrows indicates whether there is an increase (blue) or decrease (red) of the indicated parameter. **(A)** Cochlear Hcy metabolism in control mice. **(B)** Effects of ω-3 supplementation on cochlear protein levels of enzymes involved in Hcy metabolism. **(C)** Effects of folate deficiency (FD) on cochlear protein levels of enzymes involved in Hcy metabolism.

Human studies have also provided evidences of the putative prevention of SNHL by dietary supplementation with different levels of n-3 polyunsaturated (ω3) fatty acids (Rosen et al., 1970; Dullemeijer et al., 2010; Gopinath et al., 2010; Stefanutti et al., 2013). Fish oil supplementation resulted in an inverse correlation between the ingested levels of long-chain ω3 fatty acids and SNHL (Dullemeijer et al., 2010; Gopinath et al., 2010). Moreover, Curhan et al. (2014) demonstrated that regular fish consumption (2 or more servings of fish per week) and higher intake of ω3 are associated with a lower risk of HL in women, but had opposite effects on tHcy (Piolot et al., 2003; Huang et al., 2011). Dissimilar results on tHcy and/or hearing thresholds were also obtained from animal studies of ω3-suplementation carried out for limited time periods in adults or during pregnancy and lactation (Church et al., 2007, 2009, 2010; Kulkarni et al., 2011; Huang et al., 2013). A recent study focused on analyzing the effects of long-term administration of a polyunsaturated fatty acid (PUFA)-rich diet on cochlear Hcy metabolism showed limited SNHL prevention with no changes in serum folate levels or tHcy (Martínez-Vega et al., 2015b; Figure 2). Nevertheless, this diet prevented changes in cochlear expression of age-induced Hcy metabolism genes (*Bhmt* and *Cbs*) and, importantly, the altered expression of proinflammatory cytokines was precluded, and the cochlear cytoarchitecture maintained. However, there was an increase in BHMT protein levels, an enzyme involved not only in the conservation of methionine levels, but also in recycling the choline derived from phospholipid metabolism (Martínez-Vega et al., 2015b).

Altogether, these data could suggest that the diet supplemented with ω3 induces a moderate increase in intracellular Hcy levels, although the lack of significant alterations in cochlear homocysteinylated protein levels points to other more plausible possibilities. Namely, the increase in CBS levels observed with age may be necessary for the production of H_2_S, which is a regulator of the synthesis of anti- and pro-oxidant enzymes and mediators of inflammation (Ingenbleek and Kimura, 2013; Kabil et al., 2014a,b). In fact, it is known that various synthetic drugs, which supply this gas, have been used for the treatment of deafness with variable results (Lamm and Arnold, 1998; Mahmood et al., 2014). CBS also contributes to cysteine synthesis, a semi-essential proteinogenic amino acid that is mostly obtained from the diet although a 50% of that synthesized through transuslfuration is used in GSH synthesis. On the other hand, the increase of BHMT after supplementation seems to derive from the need to reduce/regulate betaine levels. Hydrolysis of phosphatidylcholine may lead, on one side, to fatty acids for the production of anti-inflammatory mediators and on the other to choline. This choline excess might be oxidized to produce the osmolyte betaine (Zeisel et al., 1980; Porter et al., 1993), and hence the need to regulate its concentrations through Hcy remethylation. As a result, the methionine cycle would recover one of the methylation equivalents used by PEMT for phosphatidylcholine synthesis, while methionine and AdoMet levels would be sustained. This possibility is supported by data obtained in the liver of animals supplemented with PUFAs, where increased expression of *Pemt* and *Gnmt* is detected; two methyltransferases expressed in the cochlea according to microarray data (Gene Expression Omnibus GSE11821; Sanchez-Calderon et al., 2010). Nevertheless, the limited cochlear effect of PUFAs supplementation was attributed to the use of a formulation especially rich in eicosapentaenoic acid, which is known to be more effective for reducing inflammation (Bhattacharya et al., 2007).

Recently, Brown et al. (2014) investigated the putative protection against noise injury exerted by the administration of nicotinamide riboside, a precursor of NAD^+^, a cofactor that is involved in the regulation of sirtuins. Defects in the function of mitochondrial SIRT3 deacetylase lead to generation of reactive oxygen species (He et al., 2012) and decreased GSH levels (Someya et al., 2010). Both are factors associated to susceptibility to noise-induced HL (Someya et al., 2010) and to the reduction of cochlear NAD^+^ levels (Ohlemiller et al., 1999). Administration of nicotinamide riboside injected twice daily for 5 days prior to noise exposure and for 48 h thereafter until sacrifice, prevented noise-induced HL and spiral ganglia neurite degeneration. These effects were mediated by the NAD^+^-dependent SIRT3, since deletion of this gene abrogated protection by nicotinamide riboside and expression of NAD^+^ biosynthetic enzymes, whereas SIRT3-overexpressing mice became resistant to noise-induced HL (Brown et al., 2014). Interestingly, AHCY requires NAD^+^ for activity, thus the use of this compound may also contribute to improve cochlear Hcy metabolism, an aspect that was not addressed by the authors and that requires further attention.

## Conclusions

Here we review recent evidence supporting the concept that the onset and progression of SNHL are closely linked to the availability of nutrients and their metabolism. Cumulative epidemiological, genetic and experimental models studies point to Hcy metabolism as a central node in the response to otic damage, although much more work is needed to fully understand cochlear Hcy metabolism, how it is regulated by nutrition, impacted by aging or noxious stimuli. However, available data fully support the potential of nutritional therapy for the protection of HL progression.

## Author Contributions

TP and IV-N, drafted the manuscript; NV, GV-M and MAP reviewed the manuscript; All authors gave final approval for publication.

## Funding

This work was supported by the European Commission FP7-PEOPLE-2013-IAPP TARGEAR and Spanish Ministerio de Economía y Competitividad (MINECO)/FEDER SAF2014-53979-R to IV-N. NV holds a CSIC predoctoral contract associated to FP7-AFHELO.

## Conflict of Interest Statement

The authors declare that the research was conducted in the absence of any commercial or financial relationships that could be construed as a potential conflict of interest.
